# Differential prefrontal-like deficit in children after cerebellar astrocytoma and medulloblastoma tumor

**DOI:** 10.1186/1744-9081-4-18

**Published:** 2008-04-15

**Authors:** Encarna Vaquero, Carlos M Gómez, Eliana A Quintero, Javier J González-Rosa, Javier Márquez

**Affiliations:** 1 Department of Experimental Psychology, Seville University, Seville, Spain; 2 Psychology Department, Neuropsychology Unit, Bosque University, Bogotá, Colombia; 3 Neurosurgery Unit, Virgen del Rocio Hospital, Seville, Spain

## Abstract

**Background:**

This study was realized thanks to the collaboration of children and adolescents who had been resected from cerebellar tumors. The medulloblastoma group (CE+, n = 7) in addition to surgery received radiation and chemotherapy. The astrocytoma group (CE, n = 13) did not receive additional treatments. Each clinical group was compared in their executive functioning with a paired control group (n = 12). The performances of the clinical groups with respect to controls were compared considering the tumor's localization (vermis or hemisphere) and the affectation (or not) of the dentate nucleus. Executive variables were correlated with the age at surgery, the time between surgery-evaluation and the resected volume.

**Methods:**

The executive functioning was assessed by means of WCST, Complex Rey Figure, Controlled Oral Word Association Test (letter and animal categories), Digits span (WISC-R verbal scale) and Stroop test. These tests are very sensitive to dorsolateral PFC and/or to medial frontal cortex functions. The scores for the non-verbal Raven IQ were also obtained. Direct scores were corrected by age and transformed in standard scores using normative data. The neuropsychological evaluation was made at 3.25 (SD = 2.74) years from surgery in CE group and at 6.47 (SD = 2.77) in CE+ group.

**Results:**

The Medulloblastoma group showed severe executive deficit (≤ 1.5 SD below normal mean) in all assessed tests, the most severe occurring in vermal patients. The Astrocytoma group also showed executive deficits in digits span, semantic fluency (animal category) and moderate to slight deficit in Stroop (word and colour) tests. In the astrocytoma group, the tumor's localization and dentate affectation showed different profile and level of impairment: moderate to slight for vermal and hemispheric patients respectively. The resected volume, age at surgery and the time between surgery-evaluation correlated with some neuropsychological executive variables.

**Conclusion:**

Results suggest a differential prefrontal-like deficit due to cerebellar lesions and/or cerebellar-frontal diaschisis, as indicate the results in astrocytoma group (without treatments), that also can be generated and/or increased by treatments in the medulloblastoma group. The need for differential rehabilitation strategies for specific clinical groups is remarked. The results are also discussed in the context of the Cerebellar Cognitive Affective Syndrome.

## Background

Posterior fossa tumors constitute two thirds of all pediatric brain tumors. The main tumors appearing in this zone are medulloblastomas, pilocytic astrocytomas and ependymomas, which together include about 90% of the cases. The astrocytoma is the most frequent tumor affecting the posterior fossa in children and in 97% of the cases it corresponds to the pilocytic astrocytoma [1,2]. Treatment must be done through surgical resection and survival can reach more than 90% in 5 years [3,4].

Medulloblastoma is a cerebellar-specific posterior fossa tumor which is very frequent in children. Its incidence is near to 50%, being the most frequent in children under 7 years old [1,4]. Treatment and prognosis are given according to age but the tumor's size, site and level of spread also must be taken into account. Survival's rate corresponds to 70% (data from 2000, [2]) after 5 years but prognosis is worse for children under 2–3 years old. Surgical resection is mandatory for treating evident tumors in all cases. Besides, in children over 4 years old, complementary treatment with radiotherapy must be applied to the neuroaxis in order to avoid a possible distant spreading, especially in patients with tumor remains after surgery or in children who present recidiva. Chemotherapy, jointly with radiotherapy, improves survival until the 60%.

One of the principal structures affected by these tumors is the cerebellum and/or its connections. The cerebellum's contribution to motor functions, coordination and balance [5-8], and to associative and non-associative motor learning [9-15] is clear. Besides, evidences of the cerebellum's relationship with associative motor learning suggested the possibility of its implication in cognitive functions [16-18]. Evidences in favor of this hypothesis have been obtained in processes as attention [19-21], language processing [22,23], short and long-term memory [24,25] and visuo-spatial ability [26]. Moreover, there are consistent findings which show that the cerebellum plays a modulatory role in frontal functions as behavioral control, modulation of affect and executive functions [21-24,27-31]. Schmahmann [31,32] has proposed a modular topography hypothesis for the cerebellum in which the vermis region would connect mainly with the orbitofrontal cortex and anterior cingulate cortex (ACC) and is related to emotional modulation and social behaviors, and the cerebellar hemispheres would connect mainly with the dorsolateral and dorsomedial prefrontal cortex (PFC) and relate to executive and cognitive processes. The evidence supporting this hypothesis comes from human and animal anatomical studies [31-37], functional neuroimaging [20,24,38-43], volumetric studies [44,45], neuropsychological studies [16,17,21,27,28,46,47] and clinical studies with patients who showed an important cerebellar dysfunction such as: schizophrenia [48], autism [19,30,47,49], olivo-ponto-cerebellar atrophy [50] and cerebellar atrophy [51].

Stuss and Benson [52] proposed that executive function could include the following processes: anticipation, goal establishment, planning, response trials, monitoring of results and use of feedback. Executive functions allow the flexible planning for execution and the continuous monitoring of information and action coordinating thoughts [53], emotions and actions in a spatio-temporal context [54] and recruiting other cognitive processes. These higher order functions allow the planning, recruitment and organization of other cognitive processes by means of initiation, modulation and inhibitory mechanisms, and participate in processes as working memory, selective attention (frontal-parietal network) and language (frontal-temporal network). About the prefrontal syndrome, Lezak [55] remarks that the loss of executive functions would affect most cognitive functions and implies loss of self-sufficiency.

The fundamental structure for executive, cognitive, behavioral and emotional regulation is the frontal cortex, which is reciprocally interconnected with polimodal associative cortex and with subcortical structures. The cerebellum's relationship to executive functions is supported by converging evidence. The neuropsychological evidence is based on the observed deficits' similarities between frontal and cerebellar lesions [27-29,49-51] and their projections [19,46,48]. The reciprocal connectivity could be in the base of these dysfunctional deficits. The cerebellum receives afferences, through the pontine nuclei, from medial and dorsolateral prefrontal cortices [36,37], posterior parietal [56], superior temporal [57,58], posterior parahippocampal [59] and from cingulate cortex [60]. Reciprocally, the crossed cerebellar-thalamic-cortical pathways project to the same cortical areas [33-35]. Recently, Dum and Strick [61] and Kelly and Strick [62], using trans-synaptic viruses tracers have mapped the connections between the prefrontal, parietal posterior and motor cortices (BA 46, 9, 7, PMv and M1) with the ventral dentate (neodentate) nucleus and have shown the reciprocal connectivity. The prefrontal area 9/46 of Petrides and Pandya [63,64] is one of the main projections to cerebellum (review in [65]). Then, cognitive and executive functions impairments observed in cerebellar patients are anatomical justified by a double feedback loop system. The Cerebellar Cognitive Affective Syndrome (CCAS) [31,32] was proposed to include the impairments in executive, linguistic, attentional, behavioral and emotional functions presented by cerebellar patients. The same authors have suggested that cerebellar modulation's loss could produce a dysmetria of thought [22,31,32,66].

Present study is part of a more broad research which tries to establish the executive and cognitive status of children suffering from cerebellum tumor resection. In a previous report [67] with the same subjects' groups (astrocytoma group: CE, medulloblastoma group: CE+ and control group: C), the clinical groups showed impairment in procedural learning while verbal declarative learning was relatively preserved. This impairment in the early phases of procedural learning was not due to a lack of basic visuomotor skills which were relatively well preserved [67]. In a forthcoming paper the attentional functions of these children will be presented. The general objective of the complete research is to establish the neuropsychological status of the clinical groups in order to facilitate rehabilitation therapies and, from a basic research point of view, to add understanding on the executive and cognitive role of the cerebellum. The specific objective of this study was the assessment of executive functions by means of neuropsychological testing in CE and CE+ groups a few years after surgery. Each clinical group was compared with a control group equated by gender, age and academic level. Only the medulloblastoma group received additional treatments (radiation- and chemo-therapy) post-surgery.

Our first aim was to detect the presence of executive and frontal functions impairments. Secondly, we tried to establish different neuropsychological profiles as a function of tumor location (vermis or hemisphere) and the affectation (or not) of the dentate nucleus. The relatively large number in the astrocytoma group (n = l3) allowed to address this objective. Finally, the influence of the resected volume, age at surgery and the time elapsed between surgery on the neuropsychological profile was assessed in order to test the dependence among executive impairments and these variables. It was also assessed the possible functional recovery as time after surgery increases.

Further, differences observed in the measured impairments could provide some clues about the implication of specific cortical regions given the different executive functions that are assessed by means of the Stroop test (incongruent condition: mainly superior medial prefrontal and anterior cingulate cortex (ACC) control [68]) and the complex Rey figure, Digit span (WISC-R subtests), Semantic and Phonologic Verbal Fluency and the WCST tests (mainly dorsolateral prefrontal cortex control) [68-71]. The applied tests are very sensitive to evaluate frontal functions and could reinforce the hypothesis of the reciprocal functional connections between the cerebellum and the prefrontal cortex needed for the executive functioning. The neuropsychological assessment would be useful in the definition of a rehabilitation strategy for these oncological patients. Finally, if differences between clinical and control groups are observed, evidence would be added to the characterization of executive dysmetria in astrocytoma and medulloblastoma cerebellar patients.

## Methods

### Subjects

Thirty-three individuals participated in this study: twenty-one of them were clinical patients and twelve were controls. The astrocytoma group (CE, without treatments) comprised thirteen individuals (ten girls and three boys), from them five had vermal tumor (11.36 ± 2.78 years) and eight had hemispheric location of the tumor (11.59 ± 3.77 years). The mean group age at evaluation was 11.50 ± 3.3 years old (range 6.66–18). The mean age at surgery was for the global CE group 8.24 ± 3.95 years (range 1.91–14.58), and, specifically, vermis CE: 8.39 ± 3.19 years and hemispheric CE: 8.15 ± 3.43 years. The mean time surgery-evaluation was: CE group: 3.25 ± 2.74 years (range 0.41–10), vermis CE: 2.97 ± 1.03 and hemispheric CE: 3.43 ± 3.49 years.

The medulloblastoma group (CE+, with treatments) comprised eight participants (three girls and five boys), but the performance of one patient was eliminated from the CE+ clinical sample. This patient suffered two surgeries due to tumor recidiva and presented a high resected volume (tumor + margin = 134.8 cc). Therefore the CE+ clinical sample was n = 7. However, it must be taken into account that the neuropsychological profile of the CE+ children, considering all 8 patients, would be worse than the one presented in this report. The mean age at evaluation was 13.59 ± 1.62 years old (range 12–17). Five children had vermis located tumor (14.11 ± 1.64 years) and two children hemispheric (12.29 ± 0.41 years). The mean age at surgery was for the global CE+ group: 7.12 ± 2.06 (range 4–9.83) and vermis CE+ group: 7.21 ± 1.44 years. The mean time surgery-evaluation was: global CE+ group: 6.47 ± 2.77 (range 2.75–10.92) and vermis CE+ group: 6.9 ± 2.69.

Variable *resected volume*. The resected tumor volume (tumor + margin) was computed following the reduced equation of ellipsoid (a × b × c/2), being a, b and c the diameters of the lesion as measured in MRI post surgery using the Volume 1.0 software (designed by University of Seville). The mean resected volume in CE group was: 49.23 ± 26.97 cc (range 7.5–122.1 cc), vermis CE: 52.97 ± 15.67 cc (range 31.4–67 cc), hemispheric CE: 46.89 ± 33.02 cc (range 7.5–122.1 cc). The mean resected volume in CE+ group was: 35.75 ± 15.73 cc (range 18–48) and vermis CE+ group 29.63 ± 16.44 cc. The data of hemispheric CE+ group (n = 2) are not presented.

Variable *dentate-affected*. The surgeon (after analysis of pre- and post-surgery MRI) categorized 6 patients as dentate affected and 7 as dentate unaffected in CE group.

In the dentate-affected subgroup there was a secondary lesion (uni or bilateral, without nucleus exeresis) due to the nucleus compression exerted by the tumor and/or the surgical operation. This circumstance appeared often related to medial and high volume tumors. In CE group the dentate-affected children were: 4 with hemisphere and 2 with vermis location.

The control group (C) comprised twelve subjects (seven girls and five boys); the mean age at evaluation was 11.33 ± 1.82 years old (range 9–14). There were no significant group differences with regard to age, sex ratio or scholarization years. Given the broad age range, the direct test scores (the values obtained by each participant) were corrected by age following normative data. Some of the clinical variables have a high variability, but this is characteristic of our patients sample collected in a two years period from the Virgen del Rocio Hospital in Seville (Spain).

The inclusion criteria for the clinical groups were: (i) normal social and emotional behavior before the diagnosis, (ii) normal academic performance before diagnosis and (iii) motor and visual performance not excessively deteriorated after surgery. The latter aspects were assessed and have been previously described in Quintero et al. [67].

The control group members were chosen among children of a private school in Seville. They were selected among a group of 50 children (from 6 to 16 years old) participating in a developmental-cognitive study who met the following criteria: standard educational opportunities, normal or corrected-to-normal visual acuity, and without any detected behavioral problems. A posteriori, one control group subject showed very low scores in the different applied tests, however we kept this subject because no uncovered pathology was detected. Table 1 summarizes the clinical groups' main characteristics.

**Table 1:** Clinical groups' main characteristics.

Histological tumor type	n = 7		
• Medulloblastoma (CE+ group)	n = 13		
• Astrocytoma (CE group)			
	**CE+ group**	**CE group**	**C group**

Location			
• Vermis	n = 5	n = 5	
• Hemisphere	n = 2	n = 8	
Neurosurgery complications			
• No	n = 3	n = 12	
• Yes (hydrocephalus)	n = 1	n = 0	
• Yes (postsurgical mutism)	n = 3	n = 1	
• Previous + others neurological sequelae	n = 2	n = 0	
Subjective deficits referred by the parents			
• No	n = 1	n = 9	
• Yes	n = 6	n = 2	
• No information	n = 0	n = 2	
Age at Evaluation (years)	13.59 ± 1.62	11.5 ± 3.3	11.33 ± 1.8
Age at Surgery	7.12 ± 2.06	8.24 ± 3.95	
Time between Surgery-Evaluation	6.47 ± 2.77	3.25 ± 2.74	
Resected Volume (cc)	35.75 ± 15.73	49.23 ± 26.97	

The present experimental protocol followed the Helsinki Declaration norms regarding experiments involving human participants and, in addition, was approved by the ethic committees of the Virgen del Rocio Hospital and the University of Seville.

### Neuropsychological instruments

We assessed some of the components referred as part of executive functions, more specifically, planning, abstraction and conceptual capacity, mental flexibility, phonologic and semantic associative verbal fluency, working memory, selective attention, response conflict, response inhibition, visual-spatial organization and construction. To fulfill this objective different highly sensitive tests were applied [55]. All tests were applied following standardized criteria. Direct scores corrected by age were transformed in T scores (normal mean = 50 ± 10) for the WCST [72], Stroop [73] and Raven-IQ [74]. Direct scores of Rey-copy test [75] were transformed in Z-scores corrected by age following Taylor scoring criteria [76]: (each subject score – mean normative data)/SD normative data. Direct scores of Digit span (WISC-R verbal scale subtests [77]) were transformed in corrected by age scaled scores (normal mean = 10 ± 3). Direct scores of Verbal Fluency test (initial letter and animal categories) were transformed in corrected by age scaled scores following the Neuropsi battery scoring criteria (Spanish-speakers children) [78].

The normal/abnormal deficit criteria considered for T and Z scores was 1.5 SD below the normative mean (severe deficit ≤ 1.5 SD, moderate to slight impairment between -1.4 SD and -1 SD). The deficit criteria considered in scaled scores (SS) was 1 SD below the normative mean (10 ± 3) because this cut-off score (7) maximize sensitivity and specificity in discriminating between normal and brain-damaged participants [72] (severe deficit ≤ 1 SD, moderate to slight impairment between -1 SD and -0.66 SD).

#### Rey/Osterrieth complex figure test

The complex Rey figure copy [75,76] was used to test planning functions, visual-spatial organization and construction. The exactness, the type and the time to complete the copy task were the variables measured. The exactness in the copy execution is a quantitative measure taking in account the number, localization and possible distortion of the reproduced elements. The type of copy is a qualitative measure (type 1 better than type 5) which is related to age. Our objective was to test the clinical groups' capacity to organize the figure's structure and the copy's planning.

#### Controlled oral word association test (F-A-S) [78]

In the Verbal Fluency test the subject was asked to say as much words as possible in a limited time. Two types of associative verbal fluency were measured: phonologic (initial letter p) and semantic (animal category) fluency. The frequency of words in one minute was measured. Additionally, the frequency of words in each quarter minute was obtained. The COWAT is a sensitive executive frontal test, but is not specific since it is also sensitive to diffuse damage [79]. This test evaluates language executive functions, but is also an indicator of speed processing.

#### Wisconsin card sorting test (WCST) [72]

This is the test most often used to assess the executive functions [52,70]. Reasoning, visual stimuli conceptualization, categorization, problem solving, set shifting, mental flexibility and self-regulation were assessed with the WCST, in which subjects have to sort cards into different piles based on changing rules. The subjects have to deduce the correct criteria for categorization (shape, colour or card number) based on the response (yes or no) of the neuropsychologist and have to produce the appropriate response. Therefore, it requires the evaluation of different possible hypothesis in order to find the correct classification rule. The percentage of conceptual responses, number of completed categories, correct responses, errors, perseverative errors and perseverative responses, are an index of conceptualization capacity, solving problems abilities, mental flexibility and self-regulation under external circumstances. The perseverative errors variable is considered an indicator of frontal lesion [80]. The WCST is particularly sensitive to different dysfunctions in frontal cortex patients [70], but does not allow discriminating between frontal lesions and frontal-pathways lesions [80].

#### Digits span (WISC-R verbal scale subtest) [77]

Digits in direct order (forward) and in inverse order (backward) require concentration and immediate memory allowing to measure the working memory capacity. Digits span backward is a more difficult task than forward because requires more executive attention in order to maintain the retained information (direct order digits) while executing the secondary task (inversing the digits order). We used the verbal digit span because is considered an excellent index of working memory. The administration, evaluation and natural direct scores (forward + backward) were corrected by age and transformed in scaled scores (normal mean 10 ± 3, cut-off: 7) following the WISC-R procedure.

#### Stroop color and word test [73]

The Stroop test is a classic executive task for measuring selective attention and the resistance to interference or inhibition of stimuli that generate automatic responses. This test also assesses conflict between competitive responses, the capacity to discard distractions, mental flexibility and self-regulation [55]. The administration of the test requires a limited time; therefore it provides a measurement of processing speed. The test presents three pages, each with 100 stimuli organized in 5 columns, and with the following tasks: (i) reading of black ink written words i.e. RED; (ii) to indicate the ink colour i.e. XXX and (iii) to indicate the colour in which the letters are written, while the word refers to another colour. Forty five seconds are allowed for each page, and the subjects have to perform as fast as possible. Administration, correction and scoring followed standard criteria. Individual direct scores corrected by age and transformed in T-scores were obtained for the three conditions: word reading, colour naming and incongruent.

#### Raven's standard progressive matrices [74]

The Raven (SPM general scale) is a normalized multiple-choice test composed of five series with 12 items without limit in execution time. This test allows assessing the conceptual capacity to establish relationships between geometrical figures in each item (spatial, numeric and geometric), perceptual abilities, visuo-spatial reasoning and intellectual non-verbal capacity. The IQ score for each child was transformed in T scores.

### Statistical analysis

We have checked that normality assumption (Kolmogorov-Smirnof with Lilliefors significance correction) was not observed in all the variables of the tests and/or different groups, so the results were analyzed using non-parametric Mann-Whitney U tests (p < 0.05) in order to compare the control group's mean performance with clinical groups. An independent comparison was performed for each clinical group and assessed measurements due to differences in the kind (cellular bases) of tumor and post-surgical treatments.

Possible influences related to the tumor's location (hemisphere or vermis) and to the affectation (or not) of the dentate nucleus on the neuropsychological performance were assessed by means of statistical analysis on the astrocytoma group (surgery without additional treatments). Mann-Whitney U test were computed to compare the performance of vermis CE group (n = 5) vs. C and hemispheric CE group (n = 8) vs. C. These comparisons would allow defining if the vermis or hemispheric located tumor was more disruptive for executive functioning. The low sample in medulloblastoma only allowed to analyze the vermis CE+ group (n = 5) but not the hemispheric CE+ group (n = 2). Additional non-parametric tests based on the Mann-Whitney U test were computed to compare dentate-affected CE group (n = 6) vs. C, dentate-unaffected CE group (n = 7) vs. C and affected vs. unaffected.

Finally, non-parametric Spearman's correlations (p < 0.05) were computed for each clinical group in order to assess the statistical dependence among the evaluated variables and the children's age at surgery, time between surgery-evaluation and resected volume.

## Results

The mean and SD of direct scores and corrected by age standard scores (T, Z and scaled scores) and the Mann-Whitney U comparison of standard scores between the groups C vs. CE+ (medullobastoma + treatments) and C vs. CE (astrocytoma without treatments) appear in table 2.

**Table 2:** Mean and standard deviations of direct (DS) and standard scores (T, SS: scaled scores and Z). Mann-Whitney U means-standard scores comparisons for the statistically significant applied tests between medulloblastoma group (at 6.47 years from surgery + with treatments) and astrocytoma group (at 3.25 years from surgery, without treatments) with the control group, respectively.

	**Control C group N = 12**	**CE group Astrocytoma N = 13**	**CE+ group Medulloblastoma N = 7**
**IQ and Executive Tests**	M ± SD Direct Scores (DS) and corrected by age T, Z and scaled score (SS)	M ± SD Mann-Whitney U	M ± SD Mann-Whitney U
**RAVEN IQ**	DS = 101.58 ± 10.35 T = 51.25 ± 6.91	DS = 93 ± 16.8 T = 45.38 ± 11.32 n.s.	DS = 85.85 ± 14.06 T = 40.71 ± 9.34 U = 14.5, **p = 0.017**
**WCST**			
**Errors**	DS = 36.83 ± 21.61 T = 48.5 ± 9.88	DS = 42.16 ± 20.16 T = 45.58 ± 8.34 n.s.	DS = 54.28 ± 16.01 T = 38.85 ± 7 U = 19, **p = 0.056 n.s.**
**Perseverative Responses**	DS = 27.66 ± 26.21 T = 46.16 ± 12.82	DS = 27.58 ± 20.04 T = 45.75 ± 10.46 n.s.	DS = 42 ± 23.07 T = 34.42 ± 10.81 U = 16, **p = 0.028**
**Perseverative Errors**	DS = 22.91 ± 19.97 T = 46.75 ± 12.60	DS = 23.83 ± 16.85 T = 45.66 ± 10.72 n.s.	DS = 33.85 ± 16.84 T = 35 ± 10.59 U = 16, **p = 0.028**
**REY COPY**			
**Exactness**	DS = 33.37 ± 3.34 Z = 0.662 ± 0.70	DS = 33.38 ± 4.01 Z = 0.764 ± 0.38 n.s.	DS = 27.28 ± 9.17 Z = -1.623 ± 2.71 U = 16, **p = 0.028**
**DIGITS (WISC-R)**			
**Verbal Digits span**	DS = 15.42 ± 2.63 SS = 9 ± 2.3	DS = 9.23 ± 3.08 SS = 5.46 ± 1.98 U = 10.5, **p = 0.003**	DS = 8.57 ± 4.72 SS = 5.42 ± 2.5 U = 6.5, **p = 0.017**
**VERBAL FLUENCY**			
**Phonologic Fluency**	DS = 12.41 ± 4.05 SS = 11.66 ± 3.6	DS = 9.76 ± 3.85 SS = 9.3 ± 3.11 U = 44.5, p = 0.06	DS = 10.42 ± 4.35 SS = 8 ± 3 U = 15, **p = 0.022**
**Semantic Fluency**	DS = 18.41 ± 3.28 SS = 11 ± 2.55	DS = 15.23 ± 4.91 SS = 8.38 ± 3.33 U = 37, **p = 0.026**	DS = 11.28 ± 4.27 SS = 4.57 ± 2.76 U = 3, **p < 0.0001**
**STROOP**			
**Word Reading**	DS = 116.50 ± 6.96 T = 54.50 ± 3.39	DS = 90.69 ± 21.11 T = 41.07 ± 10.52 U = 11, **p = 0.000**	DS = 75.33 ± 14.19 T = 33.5 ± 7.09 U = 0, **p < 0.0001**
**Colour Naming**	DS = 82.66 ± 6.38 T = 51.58 ± 4.46	DS = 65.69 ± 14.11 T = 40.23 ± 9.40 U = 20.5, **p = 0.001**	DS = 54.83 ± 767 T = 32.83 ± 5.38 U = 0, **p < 0.0001**
**Incongruent Condition**	DS = 48.25 ± 5.44 T = 53.25 ± 5.44	DS = 39.15 ± 10.87 T = 44.15 ± 10.87 U = 41, **p = 0.046**	DS = 31.33 ± 5.46 T = 36.33 ± 5.46 U = 0, **p < 0.000 1**

The CE group presented statistically significant differences with respect to controls in the three conditions of the Stroop (word: p < 0.0001, colour: p = 0.001 and incongruent: p = 0.046), digits (p = 0.003) and semantic fluency (p = 0.026) tests. The CE+ group was impaired with respect to controls in the scores of all applied tests: Stroop, three conditions: p < 0.0001, digits (p = 0.017), semantic fluency (p < 0.0001), phonologic fluency (p = 0.022), WCST-perseverative errors (p = 0.028), WCST-perseverations (p = 0.028) and Rey-copy exactness (p = 0.028). Raven-IQ showed differences too (p = 0.017).

The mean and SD of direct and corrected by age standard scores (T, Z and scaled scores) and the Mann-Whitney U (standard scores) of vermis CE, hemisphere CE and vermis CE+ groups independently compared with the C group appear in table 3.

**Table 3:** Results related to vermis and hemisphere tumor location. Mean and standard deviations of direct (DS) and standard scores (T and scaled scores: SS). Mann-Whitney U means-standard scores comparisons for the statistically significant applied tests between vermal astrocytoma, hemisphere astrocytoma and vermal medulloblastoma groups with the control group, respectively.

**Direct Scores (DS) and corrected by age T and scaled scores (SS)**	**CONTROL GROUP N = 11 Mean ± SD**	**ASTROCYTOMA VERMIS N = 5 Mean ± SD and Mann-Whitney U**	**ASTROCYTOMA HEMISPHERE N = 8 Mean ± SD and Mann-Whitney U**	**MEDULLOBLASTOMA VERMIS N = 5 Mean ± SD and Mann-Whitney U**
**Raven-IQ**	DS = 101.58 ± 10.35 T = 51.25 ± 6.91	DS = 85.40 ± 10.11 T = 40.2 ± 7 U = 6.5, **p = 0.009**	DS = 97.75 ± 18.94 T = 48.62 ± 12.67 n.s.	DS = 82 ± 15.11 T = 38.2 ± 10 U = 7.5, **p = 0.014**
**WCST Perseverat. Errors**	DS = 22.91 ± 19.97 T = 46.75 ± 12.6	DS = 25.6 ± 26.29 T = 48 ± 16.35 n.s.	DS = 22.57 ± 7.43 T = 44 ± 4.96 n.s.	DS = 37.6 ± 19.08 T = 32.4 ± 11.78 U = 10.5, **p = 0.037**
**Stroop-Word**	DS = 116.5 ± 6.96 T = 54.5 ± 3.39	DS = 92.20 ± 21.34 T = 41.8 ± 10.68 U = 13.39, **p = 0.003**	DS = 89.75 ± 22.39 T = 40.62 ± 11.13 U = 8.5, **p = 0.001**	DS = 71.5 ± 16.13 T = 31.75 ± 8.09 U = 0, **p = 0.001**
**Stroop-Colour**	DS = 82.66 ± 6.38 T = 51.58 ± 4.46	DS = 67.40 ± 18.16 T = 41.4 ± 12.32 n.s.	DS = 64.62 ± 12.23 T = 39.5 ± 7.96 U = 6, **p < 0.0001**	DS = 50.5 ± 4.79 T = 29.75 ± 3 U = 0, **p = 0.001**
**Stroop-Incongruent**	DS = 48.25 ± 5.44 T = 53.25 ± 5.44	DS = 39 ± 13.11 T = 44 ± 13.11 n.s.	DS = 39.25 ± 10.22 T = 44.25 ± 10.22 p = 0.057, n.s.	DS = 28.5 ± 4.2 T = 33.5 ± 4.2 U = 0, **p = 0.001**
**Digits span WISC-R verbal scale**	DS = 15.42 ± 2.63 SS = 9 ± 2.3	DS = 8.2 ± 1.3 SS = 4.4 ± 2.3 U = 1.5, **p = 0.005**	DS = 9.87 ± 3.75 SS = 6.12 ± 1.55 U = 9, **p = 0.029**	DS = 8 ± 5.6 SS = 5.2 ± 2.94 U = 5, **p = 0.048**
**Phonologic Fluency**	DS = 12.41 ± 4.05 SS = 11.66 ± 3.6	DS = 8.40 ± 4.03 SS = 8 ± 2.91 U = 11.5, **p = 0.048**	DS = 10.62 ± 3.73 SS = 10.12 ± 3.13 n.s.	DS = 8.8 ± 4.08 SS = 6.6 ± 2.19 U = 5.5, **p = 0.006**
**Semantic Fluency**	DS = 18.41 ± 3.28 SS = 11 ± 2.55	DS = 12.60 ± 3.04 SS = 6.4 ± 2.4 U = 3.5, **p = 0.002**	DS = 16.87 ± 5.30 SS = 9.62 ± 3.33 n.s.	DS = 11.4 ± 3.84 SS = 4.4 ± 1.81 U = 0, **p < 0.0001**

The vermis CE+ group was more impaired than the whole medulloblastoma group. However in some of the WCST variables no statistically significant differences were obtained due to the poor performance of one control subject who reduced about 3 points the mean scores of control group (the scores for this control subject were: categories: 2, errors: -1.8 SD, perseverations: < -3 SD, perseverative errors: -2.8 SD, conceptual responses: -1.9 SD below the normal mean). In spite of that, the vermis CE+ group showed statistically significant differences vs. controls in perseverative errors (p = 0.037) (table 3) and the following T-scores in errors: 38.2, perseverations: 32.2 and conceptual responses: 38.4.

The vermis CE group showed higher and different impairment profile than the hemispheric CE group compared respectively with controls. This group was statistically significantly different in the Stroop-word condition (p = 0.003), digits span (p = 0.005), semantic fluency (p = 0.002), phonologic fluency (p = 0.048) and Raven-IQ (p = 0.009). The hemispheric CE group only presented statistically significant differences in the Stroop (word: p = 0.001 and colour: p < 0.0001) and digits span (p = 0.029) tests.

The number of participants in the astrocytoma group (n = 13) allowed to perform complementary analysis to precise possible influences of dentate nucleus affectation in the evaluation (table 4). The surgeon categorized 6 patients as dentate affected and 7 as dentate unaffected. When compared these groups with C group, both presented statistically significant differences in the Stroop (word and colour) and digits span, but only the dentate-affected group showed statistically significant differences in semantic fluency (p = 0.001). The comparison between dentate-affected vs. dentate-unaffected groups showed a statistically significant higher resected volume (p = 0.035) and WCST-errors (p = 0.026) in dentate-affected patients with respect to the non-affected dentate group.

**Table 4:** Astrocytoma patients grouped by affectation (or not) of dentate nucleus at 3.25 years from surgery. Mean and SD standard scores (T and scaled scores: SS). Mann-Whitney U standard mean-scores statistically significant comparisons between: dentate-affected, dentate-unaffected patients *versus *control group, respectively. Dentate-affected compared with dentate-unaffected patients. Hemisphere dentate-affected compared with unaffected patients.

**ASTROCYTOMA GROUP **(n = 13)			
	**Dentate-affected (n = 6) *vs *C group**	**Dentate-unaffected (n = 7) *vs *C group**	**C group (n = 12)**

	Mean ± SD Mann-Whitney U	Mean ± SD Mann-Whitney U	Mean ± SD
**Stroop-word**	T = 39.66 ± 11.21 U = 2.5, **p < 0.0001**	T = 42.28 ± 10.62 U = 8.5, **p = 0.003**	T = 54.5 ± 3.39
**Stroop-colour**	T = 43 ± 8.67 U = 11.5, **p = 0.018**	T = 37.85 ± 10 U = 9, **p = 0.004**	T = 51.58 ± 4.46
**Digits span**	SS = 4.66 ± 2.06 U = 2, **p = 0.005**	SS = 6.14 ± 1.77 U = 8.5, **p = 0.038**	SS = 9 ± 2.3
**Semantic Fluency**	SS = 7 ± 1.4 U = 3.5, **p = 0.001**	SS = 9.57 ± 4.11 n.s.	SS = 11 ± 2.55
	**Dentate affected *versus *Dentate unaffected**		
**Resected volume**	63.31 ± 30.48 cc	37.16 ± 17.65 cc	U = 6.5, **p = 0.035**
**WCST-Errors**	T = 40.83 ± 5.84	T = 50.33 ± 8.05	U = 4.5, **p = 0.026**
**WCST-Perseverations**	T = 40 ± 6.87	T = 51.5 ± 10.69	trend to signif
**WCST-Persever Errors**	T = 39.66 ± 1.83	T = 51.66 ± 10.8	trend to signif
	**Hemispheric CE patients Dentate affected (n = 4) *versus *Dentate unaffected (n = 4)**		
**Semantic Fluency**	SS = 7 ± 0.81	SS = 12.25 ± 2.62	U = 0, **p = 0.029**
**Digits span**	SS = 5.25 ± 1.25	SS = 7 ± 1.41	n.s.

Following MRI data, from the six children dentate-affected, four of them had hemisphere location and two vermis location (table 4). A last comparison was done between hemispheric CE patients: dentate-affected (n = 4) vs. unaffected (n = 4). This comparison determined that dentate-affected patients showed impairment in semantic fluency (p = 0.029), but not hemispheric dentate-unaffected patients.

The table 5 shows the number of SD below the mean of the different clinical groups compared with the C group and with the normative data. The level of impairment was higher when the clinical groups were compared with controls than with population norms, except for the WCST due to the problem previously described.

**Table 5:** Level of deficit compared with normative data, and compared with control group. Number of standard deviations (SD) below the normal T-score mean (50 ± 10) and below the normal scaled-score mean (SS = 10 ± 3, cut-off: 7). Deficit criteria for T scores: -1.5 SD below normal mean and moderate to slight deficit about -1.4 SD and -1 SD. Deficit criteria for scaled scores: -1 SD below normal mean and moderate to slight deficit about -0.9 SD and -0.66 SD below normal mean.

**Executive tests**	**Global CE+ n = 7 compared with**		**CE+ Vermis n = 5 compared with**		**Global CE n = 13 Compared with**		**CE Vermis n = 5 compared with**		**CE Hemisph n = 8 compared with**	
	**C**	**norms**	**C**	**norms**	**C**	**norms**	**C**	**norms**	**C**	**Norms**

**Stroop-Word**	-2.1	-1,65	-2.27	-1.82	-1,35	-0.9	-1.27	-0.82	-1.38	-0.94
**Stroop-Colour**	-1.87	-1.71	-2.18	-2	-1.13	-0.97	-1	-0.86	-1.2	-1.05
**Stroop-incongr.**	-1.69	-1.37	-1.97	-1.65	-0.91	-0.58	-0.92	-0.6	-0.9	-0.57
**Digits span Deficit ≤ -1 SD**	-1.55	-1.53 cut-off: 7	-1.65	-1.6 cut-off: 7	-1.54	-1.51 cut-off: 7	-2	-1.86 cut-off: 7	-1.25	-1.29 cut-off: 7
**Semantic Fluency Deficit ≤ -1 SD**	-2,52	-1.81 cut-off: 7	-2.58	-1.87 cut-off: 7	-1	-0.54 cut-off: 7	-1.8	-1.2 cut-off: 7	-0.54	-0.12 cut-off: 7
**Phonologic Fluency Deficit ≤ -1 SD**	-1	-0.66 cut-off: 7	-1.4	-1.13 cut-off: 7	-0.65	-0.23 cut-off: 7	-1	-0.67 cut-off: 7	-0.42	0 cut-off: 7
**Rey-copy Exactness**	-2.28	-1.62	-2.27	-1.6						
**WCST-Errors**	-0.96	-1.11	-1.03	-1.18						
**WCST-Persever**	-1.2	-1.56	-1.4	-1.78						
**WCST-Persever Errors**	-1.2	-1.5	-1.43	-1.76						
**WCST-Concept**	-0.76	-0.91	-1	-1.16				-0.8		
**Raven-IQ**	**C III+**	**norms**	**C**	**norms**	**C**	**norms**	**C**	**norms**	**C**	**norms**
**G factor**	-1.05 IV	-0.93 IV	-1.3 IV	-1.18 IV	-0.6 III-	-0.46 III-	-1.1 IV	-0.98 IV	-0.26 III	-0.13 III

The non-parametric Spearman correlations results appear in table 6. In astrocytoma group the correlations related to the *Age-at-surgery *variable showed that those children with older age at surgery required lower number of intents (p = 0.003) to complete the WCST and made better Rey-copy type (p = 0.000) than younger children at surgery. The *time surgery-evaluation *variable showed that a longer time from surgery correlated positively with the number of corrects responses-WCST (p = 0.025). Finally, a higher *resected-volume *correlated positively with worse execution in phonologic (p = 0.002) and semantic (p = 0.026) fluency and with Rey-copy exactness (p = 0.020).

**Table 6:** Spearman's correlations related to resected volume (tumor + margin in cc), age at surgery and time elapsed between surgery and evaluation variables and all assessed neuropsychological variables for the astrocytoma and medulloblastoma groups.

**ASTROCYTOMA (n = 13) Spearman Correlations**	**RESECTED VOLUME **(mean = 49.23 ± 26.97 cc)
**Phonologic Fluency**	-0.744, **p = 0.002**
**Semantic Fluency**	-0.551, **p = 0,026**
**Rey-Exactness**	-0.572, **p = 0.020**
**Dentate nucleus affected**	0.602, **p = 0.015**
	
**ASTROCYTOMA (n = 13) Spearman Correlations**	**AGE AT SURGERY **(mean = 8.24 ± 3.95)
**WCST-Intents**	-0.732, **p = 0.003**
**Rey-copy type **(type 1 better than type 5)	-0.864, **p < 0.0001 **(negative correlation: better copy)
	
**ASTROCYTOMA (n = 13) Spearman Correlations**	**TIME surgery-evaluation **(mean = 3.25 ± 2.74)
**WCST-Correct Responses**	0.577, **p = 0.025**
	
**MEDULLOBLASTOMA (n = 7) Spearman Correlations**	**AGE AT SURGERY **(mean = 7.12 ± 2.06)
**Semantic Fluency**	0.721, **p = 0.034**

In Medulloblastoma group the age at surgery correlated positively with the semantic fluency (p = 0.034). The other variables did not showed statistically significant correlations.

## Discussion

As it can be appreciated in the tables 2, 3 and 4 the clinical groups present a differential level of impairment with respect to controls and normative data (table 5).

At 6.47 years from surgery, the medulloblastoma group compared with norms showed severe executive deficit in semantic verbal fluency (-1.81 SD, cut-off 7), digit span (-1.53 SD, cut-off 7) Stroop (three conditions: -1.65, -1.71 and -1.37 below normal mean), Rey-copy exactness (-1.62 SD), perseverations-WCST (-1.56 SD) and perseverative errors-WCST (-1.5 SD) and a moderate to slight level of impairment in errors (-1.11 SD), conceptual-WCST responses (-0.91 SD) and phonologic fluency (-0.66 SD, cut-off 7) indicating a frontal deficit. These deficits were more severe in the vermal CE+ group (table 5). However, the non-verbal Raven IQ was normal-low (-0.93 SD below normal mean, g factor IV), and more impaired in the vermal CE+ group (-1.3 SD). We want to remind here that these scores compared with C group would be worse if the outlier control subject would have been removed. Botez [27-29] has indicated that cerebellar lesions affect three main neuropsychological aspects: visuo-spatial organization, executive functions for planning and programming activities and increased RTs for visual and auditory targets, indicating a reduced information processing speed.

At 3.25 years from surgery, the astrocytoma group showed differential profile and level of impairment related to tumor location. Vermis CE patients compared with norms showed executive deficit in verbal working memory (digit span: -1.86 SD, cut-off 7) and semantic fluency (-1.2 SD, cut-off 7), moderate impairment in phonologic fluency (mean SS = 8), and about -0.9 SD below normal mean in the Stroop (word and colour), conceptual-WCST and Raven IQ (-1 SD, g IV). These results suggest that these children would present an executive deficit more marked in the language frontal-temporal network and, according to Stroop results, a reduced speed processing. When compared with the control group these scores were even worse (tables 3 and 5). The hemispheric CE group also showed deficit in working memory (digit span: -1.29 SD) and an impairment about -1 SD below normal mean in the word and colour Stroop-conditions, but interference was not impaired (-0.6 SD). We discard a possible motor origin behind the slowed speed processing because we already proved that this group did not show visuo-motor impairment as measured by the finger-tapping task, simple reaction-time (RTs) and grooved pegboard tests [67]. Only the dentate-affected subgroup of the hemispheric CE patients presented semantic fluency impairment. The phonologic fluency and the Raven IQ were normal (g factor III).

The greater impairment of the CE+ group could be due to the additive effect of the kind of tumor, pre-surgical, surgical and post-surgical problems and treatments [81,82]. It has been found that radiotherapy can affect the neuropsychological performance of children with medulloblastoma, and it is difficult to disentangle the relative contribution of radiation and the tumor's resection. Infants and very young children with medulloblastoma remain a difficult therapeutical challenge because they have the most virulent form of the disease and are at highest risk for treatment-related sequelae [81]. However, the children of the astrocytoma group did not receive chemo and/or radiotherapy but they also showed executive impairments, particularly when the tumor presented a vermal location.

As a conclusion, vermal medulloblastoma patients showed the most severe executive deficit of all tested functions, global medulloblastoma group showed severe deficit and vermal and hemispheric astrocytoma patients showed moderate to slight executive deficit and slowness in the tested functions.

### Stroop test

Attention and inhibition are important for reducing the interference of irrelevant information on the working memory, but also for suppressing previously relevant information that becomes irrelevant during the task. The impairment on inhibition also can be particularly important for decision-making in social and emotional behaviors indicating the lack of self-regulation. Frontal patients and other groups with frontal-subcortical pathways impairments present a poor execution in the Stroop tasks. Patients with right superior medial-frontal lesions (8B, 9, and superior portion of 32 BA) are the most impaired (errors and slowness) for the incongruent Stroop condition [68].

In our sample, the CE+ group compared with norms presented a clear deficit in accomplishing the Stroop tasks indicating a selective attentional deficit [19,30], poor mental flexibility and a lack of inhibitory control on interfering stimuli [83].

The CE group (vermis and hemispheric patients) compared with norms showed scores about -1 SD below normal mean for the word and colour conditions but not for the incongruent condition (-0.6 SD), suggesting that the impairment was caused by speed processing slowness. In a posterior fossa tumors patients study [84] moderate impairments in the three Stroop conditions (W = -1.25, C = -1.37 and I = -1.19) were obtained in patients evaluated at 7.5 years from surgery (surgery at 8.25 years old), but they analyzed together diverse tumor patients (most of them astrocytoma). If we collapsed the CE and CE+ groups the results would be similar to those previously described [84]. Our results are also coherent with another study [85] where it is compared the performance of young adults (23 years old) resected of astrocytoma (T ≤ 40) with a group of medulloblastoma patients with chemo- and radiotherapy (T ≤ 33).

Several functional neuroimaging studies have demonstrated the activation of the superior medial prefrontal cortex [86,87], the anterior cingulate cortex (ACC) [41,83,88,89] and the cerebellum [39,90,91] during the performance of the Stroop incongruent condition. A dysfunction in adults with cerebellar damage in the interference was shown using PET neuroimage techniques by Fiez et al. [39]. The most characteristic medial prefrontal dysfunction is the impairment of inhibitory cognitive control (mainly superior medial PFC), but other behavioral disturbances as lack of self-control and affective deficits (behavioral and emotional inhibitory control) are also present (mainly inferior medial PFC). Anterior cingulate cortex (ACC) patients present attentional deficit, distractibility, lack of initiative, apathy and flattened affect. The ACC cortex is a wide region classically related to emotions that has been cytoarchitectonically divided in different regions with sub-regions [92]. The cerebellum connectivity with these frontal regions via thalamus (parafascicular, centromedian intralaminar, dorsomedial, anterior ventral and lateral ventral nuclei) [93] would explain the Stroop deficit in cerebellar patients. In accordance with the triple attentional system described by Posner [94], the ACC is active when a conflict is present between simultaneous processes [95] as occurs in the Stroop incongruent condition, but this is not its unique function, the ACC also coordinates multiple attentional subsystems and plays a crucial role in other cognitively demanding tasks [89].

### Verbal fluency

Phonologic and semantic fluency are measurements of associative verbal fluency in restricted situations. Both verbal fluencies require the generation of words based in categories (phonological or semantical) and both require the language executive function in order to establish a search strategy, search of information, retrieval from stored information in long-term memory and active maintenance in the working memory, as well as self-regulation for an efficient performance. The frontal cortex participates in the executive control of processes as initiation, search and active evocation of phonologic and semantic representations. These last are more distributed and therefore should be more affected than phonologic fluency if executive functions are disturbed. The frontal cortex must also monitor intrusions and perseverations. Subjects with prefrontal lesions have a preserved language but show an important disconnection between knowledge and execution, and are impaired in both phonologic and semantic fluency. Lesions studies have shown that patients with left dorsolateral and striatal lesions were the most impaired in phonologic fluency performance (right dorsolateral or inferior medial frontal lesions patients were not significantly affected). Bilateral dorsolateral and (superior and inferior) medial frontal cortices are needed for an adequate performance on semantic fluency task (Stuss et al. [69] for review). Another unilateral prefrontal lesions study [96] also showed that phonological fluency is mediated by the left dorsolateral PFC. In contrast, the semantic fluency involves bilateral dorsolateral PFC and the right ventromedial frontal areas. Several functional neuroimage studies [38,90,97-99] have found cerebellar activation in verbal fluency tasks. In addition, it has been obtained an association between the cognitive demand in the semantic task and bilateral cerebellum activation [100]. Ravnkilde et al. [90], by means of PET in normal subjects, showed that during phonological fluency task left dorsolateral prefrontal cortex, left and right inferior frontal cortex, left supplementary motor cortex, left and middle anterior cingulate gyrus, left orbitofrontal cortex, right posterior temporal lobe and cerebellum (mainly in the right hemisphere) were activated.

Our cerebellar patients showed differential verbal fluency impairment. The medulloblastoma group scaled scores (SS) compared with norms (deficit -1 SD, cut-off 7) presented severe deficit (-1.81 SD) in semantic fluency and moderate to slight deficit in phonologic fluency (-0.66 SD, SS = 8). These scores are more severe in vermis CE+ patients (SF: -1.87 SD and PF: -1.13 SD). The astrocytoma group showed different profile in vermal and hemispheric patients. Vermis CE patients showed deficit in semantic fluency (-1.2 SD) and moderate impairment (-0.67 SD) in phonologic fluency. The hemispheric CE dentate-affected patients showed deficit in semantic fluency (-1 SD) but not the unaffected patients, and both had normal scores in phonologic fluency. The absence of this deficit in dentate-unaffected hemispheric CE patients, rules out the possibility that the observed deficit in vermal CE patients would be due to motor-speech impairment. Given that CE patients did not receive chemo and/or radiotherapy, the semantic fluency deficit could be due to the dysfunctional crossed cerebellum-cortical diaschisis and suggests a greater impairment in vermal CE patients and in all dentate-affected CE patients. Verbal fluency deficits have also been reported in other cerebellar patients [25,46,47,51]. For the CE+ group it could be argued that the deficit in verbal fluency is due to the radiation and/or chemotherapy. However, Riva and Giorgi [101] assessed a resected hemisphere-astrocytoma group and a vermal medulloblastoma group before receiving any additional treatment (5–6 weeks after surgery) and they reported deficit in verbal fluency in both groups.

### Digits span (WISC-R verbal scale subtests)

The first Digit span task allows testing the number of items maintained in the short-term-memory (STM). Therefore, it allows giving an estimation of the limited retention capacity in the STM and its temporal dimension (digit forward: "say the same numbers as rapid as you can"). The second task (working memory) implies an increased selective attention and executive control for the planning, allocation of attentional resources and the necessary attentional maintenance in order to interfere with the previous retention memory process when coordinates/synchronizes a verbal information (digit backward: "say the same numbers in inverse order as rapid as you can"). This subtle working memory task requires executive control, initial phonological encoding, sub-vocal rehearsal of items that are actively remembered, but also attentional focus and shifting between previous items and the on-line task (mainly parietal posterior contribution).

The multiple-component-model [102] considers two separate systems: the *phonological loop *for verbal information and the *visuospatial sketchpad *for non-verbal information, and a separate attentional controller or *central executive*. The verbal system is subdivided in a passive *phonological store *that retains some time (decay) verbal information through phonological representations, and an active *rehearsal articulatory process *that maintains by reactivation these representations.

Some functional neuroimage studies using specific neuropsychological tasks to test the Baddeley's model and other models showed differences between recognition (parietal BA 7 and temporal-parietal, supported by converging psychophysiological evidence) and recall (frontal). [103]. Lesional studies showed that posterior lesions, especially perisylvian (left ventral supramarginal gyrus) brain-damage patients are the most impaired in verbal working memory, while Broca's area patients showed moderate deficit in verbal span and severe deficit in overt or covert rehearsal working memory process, linked to speech deficit by *concurrent articulation *effect [103]. Probably, the language comprehension, symbolic abstraction and conceptualization capacities are necessary to more demanding tasks (i.e. "in inverse order").

Functional neuroimage studies [24,39,43,98,104,105] provide anatomical working memory process support in bilateral dorsolateral PFC (BA 9, 46), ventrolateral PFC (Broca: 44/45), medial frontal pre-SMA (BA 6) and ACC (BA 32) and superior posterior parietal cortices (BA 7), all related to updating information and maintaining sequential order, and bilateral inferior parietal (supramarginal gyrus BA 40) related to specific representations. Paulesu et al. [105] located the phonological store in the left supramarginal gyrus and the articulatory control process in Broca's area. Lateral cerebellar areas are also active during verbal working memory tasks [39,98,99]. Desmond and Fiez [98], by means of f-MRI, during a verbal working memory task showed that HVI and superior HVIIA cerebellar activations represent input from the articulatory control system of working memory from the frontal lobes, and that HVIIB activation is derived from the phonological store in temporal and parietal regions.

In our report, both clinical groups showed deficit in language working memory as measured by the WISC-R digit span. Medulloblastoma group showed deficit (-1.53 SD), most increased in vermal CE+ patients (-1.6 SD). Astrocytoma group children did not present evident dysartrhia problems (most related to overt or covert articulatory rehearsal). Vermal CE patients showed the most severe deficit (-1.86 SD below normal mean: 10 ± 3) in verbal working memory. These patients also showed the already described deficit in semantic fluency and moderate to slight problems in phonologic fluency, reading, naming, conceptual responses and non-verbal Raven-IQ, but the interference was less compromised (-0.6 SD), suggesting that this severe deficit in verbal working memory capacity would interfere with the correct performance in other language functions.

All hemispheric CE patients showed deficit (-1.29 SD), but it is more severe in dentate affected patients (-1.58 SD) – who also presented semantic fluency deficit – than in dentate unaffected patients (-1 SD). Digit span deficit in cerebellar tumors patients also has been reported [84,106].

The astrocytoma group impairment suggests that preservation of reciprocal cerebellar-dorsolateral PFC pathways is necessary for correct verbal executive functioning, although the specific systemic participation of the cerebellum in these high level functions is not well known.

### Rey complex figure

The obtained results for the Rey-Osterrieth complex figure copy showed that only the medulloblastoma group presented deficit with respect to norms for the exactness of the copy (Z = -1.62). This result implies difficulties in the executive planning and organization of the copy and visual-spatial perception and construction. The copy task is very difficult for prefrontal patients due to the impairment in temporal sequential planning [26-29,107], and CE+ group showed other frontal-like deficit. Other studies in children with posterior fossa tumors also found this deficit [84] showing a similar Z-score = -1.9 and suggest that the impairment is more associated to a deficit in executive functioning than to visuo-spatial problems. Patients in representative case studies [106] showed deficit too. Our results are also in agreement with those of [85], who compare the performance of medulloblastoma vs astrocytoma groups in the Rey figure copy and showed that medulloblastoma group presented a statistically significant impairment with respect to the astrocytoma group.

### Wisconsin card sorting test

The medulloblastoma group compared with norms showed deficit in perseverative responses (-1.56 SD below normal mean), perseverative errors (-1.5 SD) and moderate impairment in errors and conceptual responses about -1 SD below normal mean. Vermal CE+ patients presented a more severe deficit (perseverations: -1.78 SD, perseverative errors: -1.76 SD, errors: -1.18 SD and conceptual responses: -1.16 SD below normal mean). These results indicate difficulties with planning, cognitive flexibility, set shifting and integration of cognitive responses which are assessed by the WCST. Since frontal patients show impairment of these capacities [52,70,71], our results in CE+ group indicate a frontal-like deficit [80]. The increased number of perseverative errors and perseverations indicate deficit in the shifting strategy and the increased errors a moderate impairment in maintaining the strategy too.

In vermal-medulloblastoma subgroups at 5–6 weeks after surgery, without treatments, Riva and Giorgi [101] showed the following Z-scores for perseverations-WCST (speech-problem group = 0.5, language-problem group = 2.8, behavioral alteration group = 1.3 and one patient autistic-like = 2.4). When averaging as a global medulloblastoma group they obtained Z = 1.75, which is the same deficit level (-1.78 SD below T-normal mean) we obtained at 6.47 years from surgery and treatments.

In the astrocytoma group (at 3.25 years from surgery), dentate-affected compared with unaffected patients showed an impairment about -1 SD in perseverative errors, perseverations and errors-WCST (table 4).

The WCST is particularly sensitive to different dysfunctions in dorsolateral (left and right) and superior medial frontal cortex patients in lesion studies [70], and f-MRI studies showed the dorsolateral PFC particularly involved while solving the WCST [108,109]. The CE+ group deficit in this task must be due to cerebellar-dorsolateral connections damage caused by surgery (as in Riva and Giorgi [101]) and increased by post-surgery treatments, given that we also obtained deficit in perseverative error. Dentate-affected CE group showed moderate executive deficit too although they did not received treatments.

### Raven test

The obtained mean-score in the CE+ group was low-normal (85.85 ± 14, -1 SD below normal mean). With respect to the lack of impairment in the global CE group it must be remarked that the non verbal Raven-IQ was -1 SD in vermal CE patients, while the hemispheric group was normal. Radiation dosimetry and hydrocephalia also could be some of the factors predicting a low IQ [110-112]. However our vermis CE group neither receive radiation nor presented hydrocephalia.

### General discussion

The neuropsychological evaluation showed a differential prefrontal-like deficit: vermal medulloblastoma patients showed the most severe executive deficit and global medulloblastoma group showed severe deficit of all tested executive functions. All astrocytoma patients showed executive deficit in verbal working memory (more increased in vermal patients) and slowing of speed processing. The vermal astrocytoma patients showed semantic fluency deficit, moderate to slight impairment in phonologic fluency, conceptualization and non-verbal Raven IQ. Hemispheric astrocytoma patients showed the global group deficits, but only dentate-affected patients showed semantic deficit. All dentate-affected (vermal and hemispheric) astrocytoma patients showed semantic fluency deficit and moderate impairment (-1 SD) in perseverative errors, perseverations and errors WCST responses.

The deficit level is clearly related with the kind of tumor (cellular bases), vermal or hemispheric cerebellar tumor's location and dentate nucleus' affectation by compression without exeresis nucleus. In the few existing studies the clinical samples were small. Those reports which considered independently the kind of tumors (cellular bases) showed a greater deficit in medulloblastoma group than in astrocytoma group. Another contribution of present report is to include by first time, according to our knowledge, the dentate affectation variable as a source of possible executive and cognitive impairment.

In the astrocytoma group, higher resected-volume is significantly correlated with dentate-affectation and worse scores in verbal fluency (phonologic and semantic) and Rey-copy exactness (table 6). The older children at surgery (*age-at-surgery *statistically significant correlations) showed better Rey-copy type and required lower number of intents to complete the WCST than younger children. Longer time elapsed between surgery and evaluation (*time surgery-evaluation *statistically significant correlations) is related to more correct responses-WCST, suggesting that it is possible some improvement in conceptualization capacity as time increases after surgery. Luria [113] termed as "Frontal Secundary Syndrome" the observed deficits in adult cerebellar-tumor patients and indicated the possibility of recovery in some patients without severe sequelae.

The only significant correlation in medulloblastoma group was related to the age at surgery. Older children at surgery showed better performance in semantic fluency than younger children. Semantic maturation occurs lately than the phonologic. Therefore, younger children (range at surgery: 4–10 years old) were most immature for semantic fluency before surgery, and consequently presented difficulties in organizing this function. Some developmental MRI-studies showed that decreased amounts of normal-appearing white matter correlated with verbal thinking, non-verbal thinking, attention and IQ [114,115] in medulloblastoma children (with treatments) compared with age and gender-matched normally developing children. Neuroectodermal tumors treatments involve conformal radiotherapy and increased radiation-dose including craniospinal irradiation (without minimizing the dose to any brain structures, including the temporal lobe). Liu et al. [116], using a more sensitive technique with high-resolution MRI and an automated cortical reconstruction technique, have found abnormal changes in the grey-matter development of these patients. They compared the normal differential developmental pattern of cortical thicker (frontal and temporal lobes) and thinner (posterior cortex) in children about 10 years. Results showed significant differences in both (cortical thickness and thinner areas) and reflect significant cortical thinning in the treated medulloblastoma children compared with normal developed children. They concluded that cortical areas undergoing development are more sensitive to the radiation-dose effects.

The present report assessed functions indicate a frontal-like syndrome with differential deficits level: very severe in vermal medulloblastoma patients, severe in global medulloblastoma group and moderate to slight in vermal, hemisphere dentate-affected and hemisphere dentate-unaffected astrocytoma patients respectively. These executive deficits cannot be attributed to the chemo- or radiotherapy exclusively, given that also appeared in children who only received surgery, although it is clear that these treatments can generate and increase the reported impairments. Therefore, the obtained executive impairments support the concept of Cerebellar Cognitive Affective Syndrome (CCAS) proposed by Schmahman and Sherman [31] as a framework to characterize the cerebellar patients, but since we only have tested some executive functions (and learning capacities [67]), we cannot add more evidence about affective functions. The results obtained in our study are coherent with the role of prediction and preparation proposed for the cerebellum [117], given that after cerebellar lesion some executive functions remains, although clearly deteriorated. Paraphrasing the notion of thought dysmetria [31,32,66], this sub-optimal functioning could be defined as an executive dysmetria.

### Limitations of the study

As in similar studies, the principal limitation is the small sample. In studies with clinical participants the presence of non-controlled variables is always a concern. In this sense it has been shown that oncologic patients can suffer depression, anxiety [118] or post-traumatic stress disorder [119]. Therefore, it is very difficult to control all the possible intervening variables and they can be considered as one of the data variability sources.

## Conclusion

The following conclusions can be drawn: i) It has been shown the systemic participation of the cerebellum in executive processes in astrocytoma (without treatments) and medulloblastoma (with chemo- and radiotherapy) cerebellar patients with tumor resection. ii) These groups presented differential deficits in the executive functions depending on the kind of tumor and post-surgical treatments. iii). The astrocytoma group presented differential level of deficit: the vermal tumor resection was more disruptive than the hemispheric lesions for executive functions. The dentate-affected group is more impaired than unaffected group. iv). The observed executive dysmetria in both clinical groups is coherent with the systemic participation of the cerebellum and reciprocal ACC and Frontal cortex connections in executive functions, and with the executive aspects of Cerebellar Cognitive Affective Syndrome after cerebellar lesion. v). The increased time post-surgery is positively correlated with a relative improvement of some cognitive functions in astrocytoma children, the most susceptible group to rehabilitation. A better knowledge about neuropsychological dysfunctions after cerebellar tumor resection would help the establishment of more specific rehabilitation strategies. In a forthcoming paper, the attentional functions impairments will be evaluated by means of different computerized tasks.

## Competing interests

The author(s) declare that they have no competing interests.

## Authors' contributions

EVC participated in the design of the study, performed the scoring-corrections, statistical analysis, discussion and drafted the manuscript. CMG conceived the study, participated in its design, and helped to draft the manuscript. EAQ participated in the design of the study and collected the clinical neuropsychological data. JJGR participated in the analysis, discussion and tables. JM helped to select the participants in the study, and medically characterized each one of the subjects. All authors read and approved the final manuscript.

## References

[B1] Ries LAG, Smith MA, Gurney JG, Linet M, Tamra T, Young JL, Bunin GR, eds (1999). Cancer incidence and survival among children and adolescents: United States SEER Program 1975–1995 NIH Pub No 99-4649.

[B2] Kleihues P, Louis DN, Scheithauer BW, Rorke LB, Reifenberger G, Burger PC, Cavenee WK (2002). The WHO classification of tumors of the nervous system. J Neuropathol Exp Neurol.

[B3] Burkhard C, Di Patre PL, Schuler D, Schuler G, Yasargil MG, Yonekawa Y, Lutolf UM, Kleihues P, Ohgaki H (2003). A population-based study of the incidence and survival rates in patients with pilocytic astrocytoma. J Neurosurg.

[B4] Duffner PK, Krischer JP, Burger PC, Cohen ME, Backstrom JW, Horowitz ME, Sanford RA, Friedman HS, Kun LE (1996). Treatment of infants with malignant gliomas: the Pediatric Oncology Group experience. J Neurooncol.

[B5] Larsell O (1937). The cerebellum: A review and interpretation. Arch Neurol Psychiatry.

[B6] Dow R (1942). The evolution and anatomy of the cerebellum. Biol Rev Camb Philos Soc.

[B7] Eccles JC, Llinás R, Sasaki K, Voorhoeve PE (1966). Interaction experiments on the responses evoked in Purkinje cells by climbing fibres. J Physiol (Lond).

[B8] Ito M (1984). The Cerebellum and Neural Control.

[B9] Gormezano I, Schneiderman N, Deaux EG, Fuentes I (1962). Nictitating membrane classical conditioning and extinction in the albino rabbit. Science.

[B10] Marr D (1969). A theory of cerebellar cortex. J Physiol.

[B11] Albus JS (1971). A theory of cerebellar function. Math Biosci.

[B12] Ito M (2002). Historical review of the significance of the cerebellum and the role of Purkinje cells in motor learning. Ann N Y Acad Sci.

[B13] Yeo CH, Hardiman MJ, Glickstein M (1985). Classical conditioning of the nictitating membrane response of the rabbit: II. Lesions of the cerebellar cortex. Exp Brain Res.

[B14] Thompson RF (1986). The neurobiology of learning and memory. Science.

[B15] Delgado-García JM, Gruart A (2002). The role of interpositus nucleus in eyelid conditioned responses. Cerebellum.

[B16] Fehrenbach RA, Wallesch CW, Claus D (1984). europsychologic findings in Friedrich's ataxia. Arch Neurol.

[B17] Botez MI, Gravel J, Attig E, Vezina JL (1985). Reversible chronic cerebellar ataxia after phenytoin intoxication: possible role of cerebellum in cognitive thought. Neurology.

[B18] Leiner HC, Leiner AL, Dow RS (1986). Does the cerebellum contribute to mental skills?. Behav Neurosci.

[B19] Courchesne E, Akshoomoff NA, Townsend J, Saitoh O (1995). A model system for the study of Attention and the Cerebellum: infantile Autism. Electroencephalogr Clin Neurophysiol Suppl.

[B20] Allen G, Buxton RB, Wong EC, Courchesne E (1997). Attentional activation of the cerebellum independent of motor involvement. Science.

[B21] Casini L, Ivry RB (1999). Effects of divided Attention on temporal processing in patients with lesions of the Cerebellum or Frontal lobe. Neuropsychology.

[B22] Schmahmann JD (1991). An emerging concept. The cerebellar contribution to higher function. Arch Neurol.

[B23] Leiner HC, Leiner AL, Dow RS (1993). Cognitive and language functions of the human cerebellum. Trends Neurosci.

[B24] Desmond JE, Gabrieli JDE, Wagner AD, Ginier BL, Glover GH (1997). Lobular patterns of Cerebellar activations in Verbal Working Memory and finger-tapping tasks as revealed by f-MRI. J Neurosci.

[B25] Appollonio IM, Grafman J, Schwartz V, Massaquoi S, Hallett M (1993). Memory in patients with cerebellar degeneration. Neurology.

[B26] Townsend J, Courchesne E, Covington J, Westerfield M, Harris NS, Lyden P, Lowry TP, Press GA (1999). Spatial attention deficits in patients with acquired or developmental cerebellar abnormality. J Neurosci.

[B27] Botez MI (1992). The neuropsychology of the cerebellum: an emerging concept. Arch Neurol.

[B28] Botez Marquard T, Leveille J, Botez MI (1994). Neuropsychological functioning in unilateral cerebellar damage. Can J Neurol Sci.

[B29] Botez-Marquard T, Bard C, Leveille J, Botez MI (2001). A severe frontal-parietal lobe syndrome following cerebellar damage. Eur J Neurol.

[B30] Courchesne E, Townsend J, Akshoomoff NA, Saitoh O, Yeung-Courchesne R, Lincoln AJ, James HE, Haas RH, Schreibman L, Lau L (1994). Impairment in shifting attention in autistic and cerebellar patients. Behav Neurosci.

[B31] Schmahmann JD, Sherman JC (1998). The cerebellar cognitive affective syndrome. Brain.

[B32] Schmahmann JD (2004). Disorders of the cerebellum: ataxia, dysmetria of thougt, and the cerebellar cognitive affective syndrome. J Neuropsychiatry Clin Neurosci.

[B33] Middleton FA, Strick PL (1994). Anatomical evidence for cerebellar and basal ganglia involvement in higher cognitive function. Science.

[B34] Middleton FA, Strick PL (2000). Basal ganglia and cerebellar loops: motor and cognitive circuits. Brain Res Brain Res Rev.

[B35] Middleton FA, Strick PL, Cummings J (2001). Revised neuroanatomy of frontal-subcortical circuits. Frontal-Subcortical circuits in psychiatric and neurological disorders.

[B36] Schmahmann JD, Pandya DN (1995). Prefrontal cortex projections to the basilar pons in rhesus monkey: implications for the cerebellar contribution to higher function. Neurosci Lett.

[B37] Schmahmann JD, Pandya DN (1997). Anatomic organization of the basilar pontine projections from prefrontal cortices in rhesus monkey. J Neurosci.

[B38] Petersen SE, Fox PT, Posner MI, Mintun M, Raichle ME (1989). Positron emission tomographic studies of the processing of single words. J Cogn Neurosci.

[B39] Fiez JA, Petersen SE, Cheney MK (1992). Impaired non-motor learning and error detection associated with cerebellar damage. Brain.

[B40] Raichle ME, Fiez JA, Videen TO, MacLeod AM, Pardo JV, Fox PT, Petersen SE (1994). Practice-related changes in human brain functional anatomy during nonmotor learning. Cereb Cortex.

[B41] Pardo JV, Pardo PJ, Janer KW, Raichle ME (1990). The anterior cingulate cortex mediates processing selection in the Stroop attentional confict paradigm. Proc Natl Acad Sci USA.

[B42] Le TH, Pardo JV, Hu X (1998). 4T-fMRI study of nonspatial shifting of selective attention: cerebellar and parietal contributions. J Neurophysiol.

[B43] Cabeza R, Nyberg L (2000). Imaging cognition II: an empirical review of 275 PET and fMRI studies. J Cogn Neurosci.

[B44] Paradiso S, Andreasen NC, O'Leary DS, Arndt S, Robinson RG (1997). Cerebellar size and cognition: correlations with IQ, verbal memory and motor dexterity. Neuropsychiatry Neuropsychol Behav Neurol.

[B45] Allin M, Matsumoto H, Santhouse AM, Nosarti C, AlAsady MH, Stewart AL, Rifkin L, Murray RM (2001). Cognitive and motor function and size of cerebellum in adolescents born very preterm. Brain.

[B46] Akshoomoff NA, Courchesne E, Press GA, Iragui V (1992). Contribution of the cerebellum to neuropsychological functioning: evidence from a case of cerebellar degenerative disorder. Neuropsychologia.

[B47] Akshoomoff NA (2005). The neuropsychology of autistic spectrum disorders. Dev Neuropsychol.

[B48] Andreasen NC, O'Leary DS, Cizadlo T, Arndt S, Rezai K, Ponto LL, Watkins GL, Hichwa RD (1996). Schizophrenia and cognitive dysmetria: a PET study of dysfunctional prefrontal thalamiccerebellar circuitry. Proc Natl Acad Sci USA.

[B49] Hill EL (2004). Executive dysfunction in autism. Trends Cogn Sci.

[B50] Berent S, Giordani B, Gilman S, Junck L, Lehtinen S, Markel DS, Boivin M, Kluin KJ, Parks R, Koeppe RA (1990). Neuropsychological changes in olivopontocerebellar atrophy. Arch Neurol.

[B51] Grafman J, Litvan I, Massaquoi S, Stewart M, Siriqu A, Hallett M (1992). Cognitive planning deficit in patients with cerebellar atrophy. Neurology.

[B52] Stuss DT, Benson DF (1986). The frontal lobes.

[B53] Luria AR, Pribran KH, Luria AR (1973). Psychophysiology of the frontal lobes. The frontal lobes and the regulation of behaviour.

[B54] Fuster JM (1989). The Prefrontal Cortex: Anatomy, Physiology and Neuropsycholgy of the Frontal Lobe.

[B55] Lezack MD (1995). Neuropsychological assessment.

[B56] Schmahmann JD, Pandya DN (1989). Anatomical investigation of projections to the basis pontis from posterior parietal association cortices in rhesus monkey. J Comp Neurol.

[B57] Brodal P (1979). The pontocerebellar projections in the rhesus monkey: an experimental study with retrograde axonal transport of horseradish peroxidase. Neuroscience.

[B58] Schmahmann JD, Pandya DN (1991). Projections to the basis pontis from the superior temporal sulcus and superior temporal region in the rhesus monkey. J Comp Neurol.

[B59] Schmahmann JD, Pandya DN (1993). Prelunate, occipitotemporal and parahippocampal projections to the basis pontis in rhesus monkey. J Comp Neurol.

[B60] Vilensky JA, Van Hoesen GW (1981). Corticopontine projections from the cingulate cortex in the rhesus monkey. Brain Res.

[B61] Dum RP, Strick PL (2003). An unfolded map of the cerebellum dentate nucleus and its projections to the cerebral cortex. J Neurophysiol.

[B62] Kelly RM, Strick PL (2003). Cerebellar loops with motor cortex and prefrontal cortex of a non human primate. J Neurosci.

[B63] Petrides M, Pandya DN (1999). Dorsolateral prefrontal cortex: comparative cytoarchitectonic analysis in the human and the macaque brain and corticocortical connection patterns. Eur J Neurosci.

[B64] Petrides M, Pandya DN (2002). Comparative cytoarchitectonic analysis of the human and the macaque ventrolateral prefrontal cortex and corticocortical connection patterns in the monkey. Eur J Neurosci.

[B65] Rammani N (2006). The primate cortico-cerebellar system anatomy and function. Nat Rev Neurosci.

[B66] Schmahmann JD (2001). The cerebellar cognitive affective syndrome: clinical correlations of the dysmetria of thought hypotesis. Int Rev Psychiatry.

[B67] Quintero EA, Gómez CM, Vaquero E, Pérez-Santamaría J, Márquez J (2006). Declarative and procedural learning in children and adolescents with posterior fossa tumours. Behav Brain Funct.

[B68] Stuss DT, Floden D, Alexander MP, Levine B, Katz D (2001). Stroop performance in focal lesion patients: dissociation of processes and frontal lobe lesion location. Neuropsychologia.

[B69] Stuss DT, Alexander MP, Hamer L, Palumbo C, Dempster R, Binns M, Levine B, Izukawa D (1998). The effects of focal anterior and posterior brain lesions on verbal fluency. J Int Neuropsychol Soc.

[B70] Stuss DT, Levine B, Alexander MP, Hong J, Palumbo C, Hamer L, Murphy KJ, Izukawa D (2000). Wisconsin Card Sorting Test performance in patients with focal frontal and posterior brain damage: effects of lesion location and test structure on separable cognitive processes. Neuropsychologia.

[B71] Stuss DT, Levine B (2002). Adult clinical neuropsychology: lessons from studies of the frontal lobes. Annu Rev Psychol.

[B72] Heaton RK, Chelune GJ, Talley JL, Kay GG, Curtiss G (1997). WCST: test de clasificación de tarjetas Wisconsin.

[B73] Golden CJ (1978). Stroop Color and Word Test: a manual for clinical and experimental uses.

[B74] Raven JC, Court JH, Raven J (1977). Manual for Raven's Progressive matrices and Vocabulary Scales.

[B75] Rey A (1994). Test de copia de una figura compleja Adaptación española.

[B76] Spreen O, Strauss E (1998). A compendium of neuropsychological tests.

[B77] Wechsler D (1974). WISC-R manual.

[B78] Ostrosky F, Ardila A, Rosselli M (1999). "Neuropsi": A brief neuropsychological test battery in Spanish with norms by age and educational level. J Int Neuropsychol Soc.

[B79] Troyer AK, Moscovitch M, Winocur G, Alexander MP, Stuss D (1998). Clustering and switching on verbal fluency: the effects of focal frontal and temporal-lobe lesions. Neuropsychologia.

[B80] Anderson SW, Damasio H, Jones RD, Tranel D (1991). Wisconsin Card Sorting Test performance as a measure of frontal lobe damage. J Clin Exp Neuropsychol.

[B81] Packer RJ, Cogen P, Vezina G, Rorke LB (1999). Medulloblastoma: clinical and biologic aspects. Neurooncol.

[B82] Spiegler BJ, Bouffet E, Greenberg ML, Rutka JT, Mabbott DJ (2004). Change in neurocognitive functioning after treatment with cranial radiation in childhood. J Clin Oncol.

[B83] Bench CJ, Frith CD, Grasby PM, Friston KJ, Paulesu E, Frackowiak RS, Dolan RJ (1993). Investigations of the functional anatomy of attention using the Stroop test. Neuropsychologia.

[B84] Steinlin M, Imfeld S, Zulauf P, Boltshauser E, Lövblad KA, Lüthy AR, Perrig W, Kaufmann F (2003). Neuropsychological long-term sequelae after posterior fossa tumour resection during childhood. Brain.

[B85] Ronning C, Sundet K, Due-Tonnessen B, Lundar T, Helseth E (2005). Persistent cognitive dysfunction secondary to cerebellar injury in patients treated for posterior fossa tumors in childhood. Pediatr Neurosurg.

[B86] Milham MP, Banich MT, Barad V (2003). Competition for priority in processing increases prefrontal cortex's involvement in top-down: an event-related fMRI study of the Stroop task. Cogn Brain Res.

[B87] Pujol J, Vendrell P, Deus J, Junqué C, Bello J, Marti-Vilat JL, Capdevila A (2001). The Effect of Medial Frontal and Posterior Parietal Demyelinating Lesions on Stroop Interference. Neuroimage.

[B88] Peterson BS, Skudlarski P, Gatenby JC, Zhang H, Anderson AW, Gore JC (1999). An fMRI study of Stroop word-color interference: Evidence for cingulate subregions subserving multiple distributed attentional systems. Biol Psychiatry.

[B89] Gruber SA, Rogowska J, Holcomb P, Soraci S, Yurgelun D (2002). Stroop Performance in Normal Control Subjects: An fMRI Study. Neuroimage.

[B90] Ravnkilde B, Videbech P, Rosenberg R, Gjedde A, Gade A (2002). Putative tests of frontal lobe function: a PET-study of brain activation during Stroop's Test and verbal fluency. J Clin Exp Neuropsychol.

[B91] Salgado P, Román F, Sánchez JP, López F, Bargalló N, Falcón C, Ramírez B, Caldú X, Martínez J (2003). Activación cerebral durante el test de Stroop en un caso de lesión cerebral focal temprana. Rev Neuro.

[B92] Vogt BA, Finch DM, Olson CR (1992). Functional heterogeneity in cingulate cortex: The anterior executive and posterior evaluative regions. Cereb Cortex.

[B93] Vogt BA (2005). Pain and emotion interactions in subregions of the cingulated gyrus. Nat Rev Neurosci.

[B94] Posner MI, Dehaene (1994). Attentional networks. Trends Neurosci.

[B95] Posner MI, DiGirolamo GJ, Parasuraman R (1998). Executive Attention: Conflict, Target detection, and Cognitive control. The attentive brain.

[B96] Szatkowska I, Grabowska A, Szymanska O (2000). Phonological and semantic fluencies are mediated by different regions of the prefrontal cortex. Acta Neurobiol Exp (Wars).

[B97] Schlösser R, Hutchinson M, Joseffer S, Rusinek H, Saarimaki A, Stevenson J, Dewey SL, Brodie JD (1998). Functional magnetic resonance imaging of human brain activity in a verbal fluency task. J Neurol Neurosurg Psychiatr.

[B98] Desmond JE, Fiez JA (1998). Neuroimaging studies of the cerebellum: Language, learning and memory. Trends Cogn Sci.

[B99] Mariën P, Engelborghs S, Fabbro F, De Deyn PP (2001). The lateralized linguistic cerebellum: a review and a new hypothesis. Brain Lang.

[B100] Seger C, Desmond JE, Glover GH, Gabrieli JD (2000). Functional magnetic resonance imaging evidence for right-hemisphere involvement in processing unusual semantic relationships. Neuropsychology.

[B101] Riva D, Giorgi C (2000). The cerebellum contributes to higher functions during development: evidence from a series of children surgically treated for posterior fossa tumours. Brain.

[B102] Baddeley AD (1986). Working memory.

[B103] Chein JM, Ravizza SM, Fiez JA (2003). Using neuroimaging to evaluate models of working memory and their implications for language processing. J Neurolinguistics.

[B104] Fiez JA, Raife EA, Balota DA, Scharwz JP, Raichle ME, Petersen SE (1996). A positron emisión tomography study of the sort-term maintenance of verbal information. J Neurosci.

[B105] Paulesu E, Frith CD, Frackowiak RS (1993). The neural correlates of of the verbal component of working memory. Nature.

[B106] Levisohn L, Cronin-Golomb A, Schmahmann JD (2000). Neuropsychological consequences of cerebellar tumor resection in children. Cerebellar cognitive affective syndrome in a paediatric population. Brain.

[B107] Carlin D, Bonerba J, Phipps M, Alexander G, Shapiro M, Grafman J (2000). Planning impairments in frontal lobe dementia and frontal lobe lesion patients. Neuropsychologia.

[B108] Berman KF, Ostrem JL, Randolph C, Gold J, Goldberg TE, Coppola R, Carson RE, Herscovitch P, Weinberger DR (1995). Physiological activation of a cortical network during performance of the Wisconsin card sorting test: a positron emission tomography study. Neuropsychologia.

[B109] Monchi O, Petrides M, Petre V, Worsley K, Dagher A (2001). Wisconsin card sorting revisited: distinct neural circuits participating in different stages of the task identified by event-related functional magnetic resonance imaging. J Neurosci.

[B110] Merchant TE, Lee H, Zhu J, Xiong X, Wheeler G, Phipps S, Boop FA, Sanford RA (2004). The effects of hydrocephalus on intelligence quotient in children with localized infratentorial ependymoma before and after focal radiation therapy. J Neurosurg.

[B111] Merchant TE, Kiehna EN, Li C, Xiong X, Mulhern RK (2005). Radiation dosimetry predicts IQ after conformal radiation therapy in pediatric patients with localized ependymoma. Int J Radiat Oncol Biol Phys.

[B112] Grill J, Viguier D, Kieffer V, Bulteau C, Sainte-Rose C, Hartmann O, Kalifa C, Dellatolas G (2004). Critical risk factors for intellectual impairment in children with posterior fossa tumors: the role of cerebellar damage. J Neurosurg.

[B113] Luria AR, Tsvetkova LS (1967). Les troubles de la resolution de problèmes.

[B114] Mulhern RK, Palmer SL, Reddick WE, Glass JO, Kun LE, Taylor J, Langston J, Gajjar A (2001). Risks of young age for selected neurocognitive deficits in medulloblastoma are associated with white matter loss. J Clin Oncol.

[B115] Reddick WE, Russell JM, Glass JO, Xiong X, Mulhern RK, Langston JW, Merchant TE, Kun LE, Gajjar A (2000). Subtle white matter volume differences in children treated for medulloblastoma with conventional or reduced dose craniospinal irradiation. Magn Reson Imaging.

[B116] Liu AK, Marcus KJ, Fischl B, Grant E, Young T, Rivkin MJ, Davis P, Tarbell NJ, Yock TI (2007). Changes in cerebral cortex of children treated for medulloblastoma. Int J Radiat Oncol Biol Phys.

[B117] Courchesne E, Allen G (1997). Prediction and preparation, fundamental functions of the cerebellum. Learn Mem.

[B118] Steinbach JP, Blaicher HP, Herrlinger U, Wick W, Nagele T, Meyermann R, Tatagiba M, Bamberg M, Dichgans J, Karnath HO, Weller M (2006). Surviving glioblastoma for more than 5 years: the patient's perspective. Neurology.

[B119] Ursano RJ, Fullerton CS (1999). Posttraumatic stress disorder: cerebellar regulation of psychological, interpersonal, and biological responses to trauma?. Psychiatry.

